# Global Trends in Research of Intestinal Cells and Type 2 Diabetes Mellitus: A Bibliometric and Visualized Analysis from 2004 to 2023

**DOI:** 10.2174/0118715303353611250312043509

**Published:** 2025-04-04

**Authors:** Zhaoyang Li, Shuang Zhang, Rufan Cao, Bo Wu, Kangning Han, Wenhui Yang, Shiyao Chen, Xinyu Zhang, Xiaochun Han, Liangqing Guo

**Affiliations:** 1 The First Clinical Medical College, Shandong University of Traditional Chinese Medicine, Jinan, 250355, China;; 2 College of Foreign Language, Shandong University of Traditional Chinese Medicine, Jinan, 250355, China;; 3 Department of Cardiovascular, Affiliated Hospital of Shandong University of Traditional Chinese Medicine, Jinan, 250011, China;; 4 College of Traditional Chinese Medicine, Shandong University of Traditional Chinese Medicine, Jinan, 250355, China;; 5 College of Health, Shandong University of Traditional Chinese Medicine, Jinan, 250355, China;; 6 Department of Endocrinology, Affiliated Hospital of Shandong University of Traditional Chinese Medicine, Jinan, 250011, China

**Keywords:** Intestinal cell, type 2 diabetes mellitus, bibliometric, CiteSpace, VOSviewer, systemic metabolic disease

## Abstract

**Background:**

As a systemic metabolic disease, type 2 diabetes has gradually become a disease that seriously threatens human health and quality of life worldwide. Many studies have shown that its pathogenesis and treatment are closely related to intestinal cells. However, the research in this field lacks relevant bibliometric analysis. This study aims to review the knowledge structure and research hot spots on intestinal cells and type 2 diabetes and provide a new direction for its future research through bibliometric methods and visual knowledge maps.

**Methods:**

Using the Web of Science Core Collection (WoSCC) database, we searched the related literature on intestinal cells and type 2 diabetes from 2004 to 2023, and made bibliometric analysis through VOSviewer, CiteSpace, the “Bibliometrix” R package, and Scimago Graphica. Combined with Bradford’s Law, Price’s Law, Lotka’s Law, and Zipf’s law, bibliometrics analysis was conducted on publication time, author, country/region, keywords, journals, and references related to publications in this field.

**Results:**

A total of 394 articles were included from 51 countries, led by the United States and China. In the past 20 years, the publication of relevant literature has shown a steady growth trend. These articles are from 2486 authors. Among them, Ti-dong Shan and Tao Yu from China have published the most literature, the University of Toronto is the main research institution, the Journal of Biological Chemistry is the journal with the most common citations, and the most prominently cited article is Sugar absorption in the industry: the role of GLUT2.

**Conclusion:**

This study is the first bibliometric analysis that comprehensively summarizes the research trends and hot spots of intestinal cells and type 2 diabetes. This information determines the research hot spots and research frontiers in this field. The hot spots of research are “intestinal epithelial cells”, “GLP-1”, “SGLT-1”, “GLUT2”, and “intestinal endocrine cell”, while “intestinal epithelial stem cells”, “short-chain fatty acid”, “bile acid” and “bariatric surgery” may be the focus of future research, which helps clinicians better understand the relationship between intestinal cells and type 2 diabetes, and provides a reference direction for future scholars in this field.

## 1 INTRODUCTION

Type 2 diabetes mellitus (T2DM) is a chronic metabolic disease caused by relative insulin deficiency [1]. With the continuous improvement of people’s living standards, its incidence rate has increased year by year worldwide [2]. As the disease progresses, it will eventually lead to serious multisystem complications, seriously affecting the quality of life of patients and imposing a huge economic burden on every country [3, 4]. Therefore, it is particularly important to understand the etiology of type 2 diabetes and formulate prevention strategies accordingly.

A large number of works of literature have revealed the possible risk factors of type 2 diabetes, including genetic factors, health status, eating habits, lifestyle and environmental factors [5-7]. With the continuous deepening of research, it has been found that mammalian intestinal cells are involved in a series of physiological processes crucial to human energy metabolism, such as metabolism, energy homeostasis, and immune activity [8, 9]. These physiological processes are closely related to the onset and treatment of type 2 diabetes. The relevant literature has been published. It can be seen that there is a close relationship between intestinal cells and type 2 diabetes. However, the current research literature and data on the pathogenesis and treatment of intestinal cells and T2DM are scattered, and bibliometrics studies are more conducive to relevant researchers to effectively grasp the main results of previous studies and refine hot spots for future prospective studies.

Although many traditional reviews and meta-analyses have conducted a large number of studies on the two, they only focus on a specific aspect, and the scope of analysis and discussion is narrow. Bibliometrics focuses on quantifying the complete body of knowledge by analyzing quantitative literature and predicting the future direction of the field of study through intuitive visualization. In order to review the current research hotspots and future research trends of the relationship between intestinal cells and type 2 diabetes comprehensively and objectively, this study used the research published in the WOS database by CiteSpace, VOSviewer, the “Bibliometrix” R package and Scimago Graphica for bibliometric analysis.

## 2 MATERIALS AND METHODS

### 2.1 The Article Source Process

We searched for literature in the WOS database from January 1, 2004 to December 31, 2023: TS = ((“intestinal cells” OR “enterocytes” OR “duodenal mucosal cells” OR “intestinal epithelial cells”) AND (“T2DM” OR “type 2 diabetes mellitus” OR “type 2 diabetes” OR “hyperglycemia” OR “hyperglycemia” OR “hyperglycemic syndrome” OR “diabetic” OR “diabetes” OR “high blood glucose” OR “high blood sugar” OR “diabetes mellitus” OR “elevated glucose blood levels”)).

A total of 529 articles were obtained. Subsequently, the content of each article (including the title and abstract of the paper) was manually reviewed to exclude irrelevant publications, withdrawn articles and non-English articles so as to ensure that the selected articles involved intestinal cells and type 2 diabetes. Duplicate articles were excluded from the study. Finally, 394 articles were retained for bibliometric mapping analysis.

### 2.2 Research Tools and Methods

Bibliometrics first emerged in the early 20^th^ century and became an independent discipline in 1969 [10, 11]. Visual knowledge graphs are an important scientific branch for exploring a certain field and a new development in bibliometrics [12]. Therefore, the development of a research field can be obtained through bibliometric analysis. Among them, CiteSpace, VOSviewer, Bibliometrix and Scimago Graphica are four powerful visual knowledge graph tools. They each have their own strengths and complement each other. CiteSpace’s Timezone and Timeline can depict the knowledge evolution process of a discipline in the time dimension, and its emergence word detection function can sort out the latest professional terms published in a certain year, which can help us see the cutting-edge research of a discipline more intuitively [13]. VOSviewer provides three visualization methods: Network visualization, Overlay visualization, and Density visualization, and reflects more intuitively the connections within the discipline [14]. Based on the same dataset, Song compared VOSviewer with CiteSpace and found that VOSviewer is more accurate in clustering algorithms, while CiteSpace performs better in rendering evolution [15]. On the other hand, Bibliometrix is an open-source software based on R package that is widely used in bibliometrics research [16]. Scimago Graphica analyzes disciplines in the form of geometric space, facilitating research in different regions. In addition to using software, this study also combined Bradford’s law, Lotka’s law, Price’s law, Zipf’s law, H-index and M-index and other indicators to further analyze the literature data. Price’s law is mainly used to determine the core group of authors [17]; Lotka’s law reveals the relationship between the number of authors and the number of papers in the research field [18]; Bradford’s law can identify the most influential journals [19]; Zipf’s law can search for more meaningful keywords in the research field [20]; H-index and M-index can be used to evaluate the academic achievements of researchers in this field [21].

## 3 RESULTS

The 394 papers used in this study were written by 2486 authors from 683 organizations situated in 51 countries, published in 262 journals, and cited 22037 references from 3121 journals.

### 3.1 Bibliometric Analysis of the Publications

Based on our selection process, a total of 394 studies on intestinal cells and T2DM have been retrieved over the past 20 years. The number and fluctuation of published articles not only reflect the prosperity of a research field but also the development prospects of that research field [22]. By collecting publication data from January 1, 2004 to December 31, 2023 (Fig. **1**), this study found that the overall number of published papers showed a fluctuating upward trend. Since 2013, the number of papers published each year has been concentrated at around 20, and after 2020, the number of papers published each year has reached over 30. In addition, to further evaluate the trend of changes in this research direction, we calculated the exponential function Y = 5.8137e^0.0982X^ (R^2^=0.7321, X is the year, Y represents the number of publications). This indicates that in recent years, the field of “intestinal cells and T2DM” has received attention and entered a period of rapid development, which may become a promising research direction in the future.

### 3.2 Bibliometric Analysis of the Author

By analyzing the author’s collaborator network, one can gain a deeper understanding of the representative scholars and core research forces in this field. According to statistics, a total of 394 papers have been published with 2486 authors. According to bibliometrix, this study complies with Lotka’s law, and Table **S1** lists the productivity of all authors who follow Lotka’s law [18].

According to Price’s Law [17], the minimum number of papers published by a core author in a certain field is m = 0.749√n_max_ = 2 (m integer, n_max_ is the number of papers published by the author with the highest number of papers, as can be seen from VOSviewer statistics with n_max_ = 8). Therefore, authors with two or more papers are positioned as core authors in that field. According to statistics, there are 152 core authors and a total of 236 papers have been published, accounting for 59.89% of the total number of publications and meeting the Price’s Law standard of 50%. Use VOSviewer to overlay and visualize the collaborative network with Total Link Strength > 2 in the core authors (Fig. **2**). In (Fig. **2**), node sizes represent the number of published papers, and node colors and connections represent partnerships. Different colors represent different clusters. We observed close collaboration among multiple authors and even close collaboration among authors in different clusters.

It can be seen that after 20 years of development, the core group of authors of “Intestinal Cells and Diabetes” has initially formed. We list authors whose number of publications is n≥5 (Table **1**). H-index combines the publication frequency and cited frequency in this research field to better reflect the influence of an author’s research results, while M-index considers the research years compared with H-index [21]. Bibliometrix calculates the H-index and M-index of the top 9 highly productive authors. Among the top 9 prolific authors, Ti-dong Shan from China contributed the most articles (N = 8). In addition, Neha Shrestha from the Research Institute for Bioscience and Biotechnology, Kathmandu, Nepal, is also the author with the highest average number of citations per paper (N = 89.6).

### 3.3 Bibliometric Analysis of the Country and Institution

Over the past 20 years, 51 countries have participated in research in this area. We screened and visualized published articles (N ≥ 5), selecting a total of 22 countries. Then, we established a visualization network for the number and publication relationship of articles in these 22 countries. The size of the area represents the number of published articles, and the depth of the color represents the strength of the association of the published article (Fig. **3**). From the graph, we can see that the top ten countries are distributed in North America, Asia, and Europe (Table **2**). Among these countries, the United States and China account for half (50%) of the total publication volume. It is worth noting that there is close cooperation between different countries, such as the United States and Japan, France, Israel, China and the United States, Germany, and Canada. In addition, we have noticed that South Korea has the top ten publications (N = 12) but has not cooperated with other countries.

In addition, a total of 683 institutions participated in this research direction, and the top 10 institutions with published papers were “University of Toronto” (N = 11), “University of Porto” (N = 8), “University of Turku” (N = 8), “Sun Yat sen University” (N = 8), “University of Helsinki” (N = 7), “University of Wurzburg” (N = 7), “German center for diabetes research” (N = 6), and “Technische Universityät”. M ünchen (N = 6), the Chinese University of Hong Kong (N = 6), Inserm (N = 6). The top ten universities are also located in North America, respectively, Asia and Europe.

### 3.4 Bibliometric Analysis of the Keywords

#### 3.4.1 Co-occurrence Analysis of the Author Keywords

A total of 394 articles included 1084 keywords. Fig. (**4**) shows the clustering analysis results generated by VOSviewer (f > 2), including dynamic changes and network diagrams. The overlay visualization adds time factors, and the color gradient can intuitively reflect the recent activities of each scholar (Fig. **4**). Node size represents the frequency of keyword occurrence, node color represents time distribution (average year of occurrence), and connectivity represents partnership. Through visual analysis of keyword overlay years, we can see the development direction of this research field, which has a great possibility of predicting future research directions [23-27]. We removed the keywords “diabetes”, “intestin”, “diabetes mellitus”, “type 2 diabetes”, “hyperglycemia”, “glucose”, “type 2 diabetes mellitus”and “small intestine”, and merged the same keywords to use reference frequency as the main observation for analysis. The top 10 high-frequency keywords include “intrinsic epithelial cells” (N = 33), “objectivity” (N = 23), “glp-1” (N = 20), “insulin” (N = 20), “caco-2 cells” (N = 14), “sglt1” (N = 13), “metformin” (N = 12), “differentiation” (N = 10), “gut microbiota” (N = 10), and “inflammation” (N = 10). Zipf’s law can screen out core keywords (Figs. **S1** and **S2**), n calculated as 32.09, so the first 32 keywords are high-frequency keywords. Table **3** shows the top 32 keywords based on Zipf’s law (Fig. **S3**) and VOSviewer of the frequency of occurrence [20].

From Fig. (**4**), it can be seen that the entire graph focuses on the two core keywords, “intestinal epithelial cells” and “insulin”, with “glp-1” forming a secondary keyword. This research direction can be roughly divided into three periods. The first period is before 2015, mainly focusing on the relationship between insulin and blood sugar. The second period is from 2016 to 2022, during which the frequency of keyword appearances is the highest. This also marks the flourishing development of research directions during this period, mainly focusing on intestinal epithelial cells, transport proteins, *etc*. The third period is from 2022 to the present, mainly focusing on microorganisms in the intestine, high-fat diets, *etc*.

#### 3.4.2 Timeline View Map Analysis of the Keywords

Although the keyword clustering density view of VOSviewer can visually display hot topics in the research field, if you want to observe the trend of keyword emergence and development in time series, you need to use CiteSpace’s time zone view to visually display the distribution of hotspots in each time period [28]. The frequency of keywords must be selected appearing from January 2004 to December 2023 (Slice Length = 3) for time zone view distribution, cluster them with related keywords, and select the top nine clusters from them. (Fig. **5**). The clustered graph obtained ideal Modularity (Q = 0.4097) and Silhouette (S = 0.7097). We can see that cluster #1 “insulin resistance” appeared earlier and reached its peak in research and publication from 2008 to 2013. However, the popularity of its cluster showed a decreasing trend over time, and its newly emerged keywords gradually disappeared in 2019. On the other hand, cluster #0 “gut microbiota” has emerged rapidly since its first appearance in 2007, and its related keywords have been increasing year by year. The links between related keywords are very close, and there is no sign of a cooling trend, making it the mainstream research direction at present.

### 3.5 Bibliometric Analysis of the Journal

Bradford’s law accurately identifies the most influential journals in the field [19]. Bradford’s Law analysis of publication sources shows that the most published journal is the International Journal of Molecular Sciences, published by MDPI (11 papers, 2.8% of total publications). This was followed by the Journal of Biological Chemistry (7 papers, 1.7% of total publications). Fig. (**S3**) shows the six journals that form the core of the most productive journals in this research topic according to the Bradford region (Table **S2**).

### 3.6 Citation Bursts Analysis of the References

To have a clearer understanding of the sudden outbreak of research hot spots in this field, “Bursts” analysis of Citespace could be adopted to present it. Citation explosion type references refer to references that are frequently cited by authors in a certain field within a certain period of time, indicating that they have received a lot of attention during this period [29]. We selected the top 25 citations with strong citation intensity and sorted them based on the time of citation. The earliest citation burst reference was in 2004, with the earliest citation burst reference published in 2002 by Mehran Haidari in August 2002 [30] and the latest citation burst reference published in 2020 by Hermann Koepsell in August 2020 [31]. The burst intensity of these 25 references ranges from 1.79 to 3.8, maintained for 2-7 years. The strongest outbreak came from George L Kellett’s article titled “Sugar absorption in the intestine: the role of GLUT2” published in the Annual Review of Nutrition in 2008 [32].

Table **4** shows the top 5 references with citation bursts arranged by length analyzed from the type of article, except for the first article, which is a review, the remaining 4 articles are all mouse clinical trials (MCTs) [32-36]. Furthermore, the main topic of the cited references is to explore how SGLT1 and GLUT2 affect the absorption of glucose by intestinal cells.

### 3.7 Bibliometric analysis of the Co-citation

#### 3.7.1 Bibliometric Analysis of the Co-citation Journal

As shown in Table **5**, among the top 10 co-cited journals, 6 journals were cited more than 400 times, Journal of Biological Chemistry (N = 900) has the highest number of co-citations, followed by diabetes (N = 703), and Q1 articles occupy eight out of the top ten. To further analyze, 44 journals with a minimum number of co-citations equal to 95 were selected, and a co-citation network diagram was drawn (Figs. **6** and **7**). As shown in the figure, The Journal of Biological Chemistry and Diabetes, The Proceedings of the National Academy of Sciences of the United States of America, Nature, American Journal Of Physiology-gastrointestinal And Liver Physiology, Gastroenterology, *etc*. have a positive co-citation relationship.

The co-citation network drawn by co-cited journals can be divided into four major clusters corresponding to the four colors in the graph. Among them, green clustering mainly refers to journals in the fields of diabetes and endocrinology; blue clustering mainly refers to journals in the field of gastrointestinal digestion; red clustering refers to journals in the field of natural science; yellow clustering refers to journals in the field of liver disease and other metabolic related fields. It can be seen that this research direction involves multiple disciplines in various fields.

The double graph overlay shows the distribution of this study relative to the main research disciplines, intuitively reflecting the research dynamics of the disciplines. The left side is a cluster of cited journals, and the right side is a cluster of cited journals. The curve between them represents the citation path, and the label represents the disciplines covered by the journal. The yellow path in Fig. (**8**) represents that research published in Molecular/Biology/Genetics journals is mainly cited by literature in Molecular/Biology/Immunology journals (z = 6.002281, f = 93104); the green citation path indicates that research in Molecular/Biology/Genetics journals is often cited by Medicine/Medical/Clinical journals (z = 1.7044213, f = 29942).

#### 3.7.2 Bibliometric Analysis of the Co-citation Author

The articles of a total of 17144 co-cited authors were frequently cited in this research direction, with 5 authors being cited more than 40 times. The most frequently cited author among them is Patrick D Cani (N = 67), followed by George L Kellett (N = 62). Fig. (**9**) shows the co-citation analysis network map by setting the minimum equal citation as 30. As shown in the figure, there is a positive collaboration among different co-cited authors, such as Patrick D Cani and Ernest M Wright, George L Kellett and Bernard Thorens.

Analysis of the co-citation network of highly co-cited authors can be divided into three major clusters, representing the three colors in the figure. The red cluster represents the authors in the field of diabetes, the blue cluster represents the authors in the field of GLP-1, and the green cluster represents the authors in the field of intestinal digestion.

## 4 DISCUSSION

This study used the WOS database to retrieve relevant literature in the field of intestinal cells and type 2 diabetes from 2004 to 2023 and adopted VOSviewer and Scimago Graphica software to conduct a visual analysis of literature data from the past 20 years to understand the research status in this field over the past 20 years. The annual publication volume, authors, countries, institutions, keywords, references, and co-cited journals and authors were quantitatively analyzed, and the research hot spots and frontiers in this field were explored.

### 4.1 General Information

From the perspective of annual publication volume (Fig. **1**), the number of publications in this field was 10 in 2004, and the annual publication volume has been gradually increasing in the past 20 years. This also indicates that this field has gradually attracted more attention and research from scholars, and its influence has been increasing year by year. Based on statistical analysis of the various countries and institutions that have published articles, it is possible to select countries and institutions with a large volume of articles and high impact and determine their cooperation relationships. Among them, the United States and China are the main countries studying this field, and except for South Korea, all other countries have established a relatively close network of connections. Among the top ten research institutions, 3 are from Germany, 2 are from China, and 2 are from Canada, with the University of Toronto publishing the most articles. Various countries and institutions have established strong cooperation and exchange networks, which are conducive to mutual communication and promotion between countries, eliminating academic barriers and breaking geographical limitations. Among the top 10 authors in terms of publication volume, there are 5 authors from China, and the top 3 authors are Ti-dong Shan, Tao Yu and Qi-kui Chen, which indicates that these three authors have made outstanding contributions to the field. Professor Hermann Koepsell retrospectively analyzed the absorption of glucose by glucose transporters located in the small intestine, and concluded that SGLT1, GLUT2 and GLUT5 in the small intestine can mediate the absorption of glucose, and diabetes may be caused by the dysfunction of small intestine transporters [37]. Po Sing Leung believed that the physiological function of the small intestine directly affected blood glucose levels and reviewed the effects of intestinal regulatory mediators on intestinal glucose absorption. The research also found that niacin can activate the nicotinic acid receptor GPR 109 a (167 a) to enhance intestinal glucose uptake in db/db T2DM mice and CaCO_2_ (epithelial colorectal adenocarcinoma) cells [38, 39]. Patricia Serradas has shown that HNF-4γ has a regulatory effect on intestinal endocrine cells, and the absence of HNF-4γ can regulate intestinal endocrine cells to improve glucose homeostasis [40]. Bruno Sarmento prevents diabetes-related fibrosis by targeting the receptor FcRn expressed in epithelial cells [41]. In conclusion, the above research mainly focuses on the pathogenesis and treatment of intestinal cells and type 2 diabetes.

In terms of co-cited journals, it could be seen that most of the co-cited journals in the top 10 are highly influential Q1 journals, and JCR zoning is a journal-specific indicator related to impact factors, rankings, and category quarters [42, 43]. Periodicals not only involve diabetes, metabolism and digestion, but also natural science, basic science and clinical medicine intertwined. Obviously, these journals are high-quality international journals with a very rich variety of disciplines, which provide strong theoretical support for our research.

In terms of co-cited authors, Patrick D Cani is the most frequently cited author, followed by George L Kellett. In 2004, through a randomized controlled trial (RCT) Patrick D Cani studied that dietary fiber can promote the release of glucagon like peptide-1 (7-36) amide by intestinal endocrine cells [44]. In the following year, Patrick D Cani also confirmed that the GLP-1 (7-36) amide of diabetes mice fed with dietary fiber was higher than that of diabetes mice fed with a normal diet, and their blood sugar and appetite were better than those of diabetes mice fed with a normal diet [45]. Subsequently, in 2007, Patrick D Cani traced the source and found that dietary fiber has an upregulation effect on neurogenic protein 3 and NeuroD, which can promote the differentiation of intestinal L cells, thereby increasing the production of endogenous GLP-1 [46]. In the same year, Patrick D Cani investigated the relationship between the secretion of intestinal proinsulin (GLP-1 and GIP) and GLUT2, which showed that the secretion of both types of intestinal proinsulin depends on the mechanisms involved in their own receptors, and GLP-1 further required GLUT2 [47]. In recent years, Patrick D Cani has made contributions to the influence of intestinal microbiota on diabetes. In short, the research achievements of Patrick D Cani have provided strong evidence for the intestinal cells on the diet of diabetes and laid a theoretical and experimental foundation.

The explosion of references illustrates emerging themes in this field in different years [29]. Sugar absorption in the intestine: the role of GLUT2 by George L Kellett, published in 2008, shows the greatest intensity and longer duration, which reviews the effect of GLUT2 on sugar absorption in the intestinal tract, clarifies the idea of GLUT2 in the impact of intestinal cells on diabetes, and makes the theory more systematic [32]. GLUT2 has remained a research hot spot in recent years. Secondly, Nima Saeidi published an article in Science in 2013, which first proposed that glucose metabolism in the intestinal tract was reprogrammed after Roux-en-Y gastric bypass (RYGB), which contributed to the improvement of blood glucose control after RYGB and provided a new theoretical basis for RYGB treatment of type 2 diabetes [36]. In addition, keywords can also help us quickly observe the hot spot distribution and evolution process of intestinal cells and type 2 diabetes.

From (Fig. **4**), we can see that most of the macromolecules studied before 2014 were insulin and other macromolecules directly related to diabetes. With the continuous updating of technology and the continuous development of disease research, glucose transporters such as SGLT-1 and GLUT2 have gradually become emerging hot spots. Obesity and other metabolic-related directions have gradually been studied by researchers. In recent years, with the continuous in-depth understanding of diabetes, people's research on diabetes has become more systematic and integrated, gradually shifting the direction to gastrointestinal and other digestive systems, and intestinal microbes have gradually become the research frontier. In addition to the main keywords such as “intestinal epithelial cells”, “obesity”, “GLP-1” and “insulin”, Table **3** also includes “SGLT-1”, “GLUT2”, “intestinal endocrine cells”, “intestinal epithelial stem cells”, “short-chain fatty acids”, “bile acids”, “bariatric surgery” and other keywords. From (Fig. **5**), we can see that insulin resistance has gradually disappeared as an emerging direction since 2019, which may indicate that research on insulin resistance has become more precise. Intestinal epithelial cells, GLP-1, SGLT-1, GLUT2, intestinal endocrine cells, from the beginning to today, still maintain a high heat and appear more frequently in Top 5 Citation Burst. We can think that intestinal epithelial cells, GLP-1, SGLT-1, GLUT2, and intestinal endocrine cells are the hot issues in current research. As high-frequency keywords, intestinal epithelial stem cells, short-chain fatty acids, bile acids, and sleeve gastrectomy were all initially introduced in 2020-2023, as shown in Fig. (**5**), and gastric bypass was also mentioned in the references with citation burst. Therefore, intestinal epithelial stem cells, short-chain fatty acids, bile acids, and gastrectomy may be the future frontier directions. Based on this, we discuss the current hot spots and future research.

### 4.2 Current Research Focus on Intestinal Cells and T2DM

#### 4.2.1 Intestinal Epithelial Cells Participate in Glucose Uptake, Transportation, and Absorption through Glucose Transporter Proteins SGLT-1 and GLUT2

The glucose transporters located in intestinal cells directly participate in regulating blood glucose stability. According to the analysis of the selected literature, 3 of the top 5 references with citation burst were related to glucose transporters (Table **4**). The glucose transporters in the body can be mainly divided into two categories according to their absorption modes: sodium glucose co-transporters (SGLT) are involved in classical active transport, and glucose transporters (GLUT) are involved in diffusion and absorption [48]. The currently developed hypoglycemic agent SGLT-2i has a significant curative effect, mainly acting on the SGLT-2 glucose transporter in the proximal convoluted tubules of the kidney. For intestinal cells, SGLT-1 and GLUT2 in the transporter family are the main glucose transporters [49-51]. Specifically, SGLT-1 is mainly located in the brush-like border membrane (BBM) of intestinal cells, and GLUT2 is mainly located in the basal lateral membrane (BLM) or BBM plus BLM at low and high levels of D-glucose concentration in the lumen. Specifically, the D-glucose absorption of low lumen glucose is mediated by SGLT1 in BBM and GLUT2 in BLM, while the high-capacity D-glucose absorption of high lumen glucose is mediated by GLUT2 in SGLT1 and BBM and GLUT2 in BLM [31]. Animal experiments have found that D-glucose is mainly mediated by SGLT1 through intestinal BBM and is not related to glucose load. High glucose concentration increased the content of SGLT1 and GLUT2 in BBM, and SGLT1 was essential for the upregulation of GLUT2 [35]. Therefore, reducing postprandial blood sugar can start by inhibiting the function of transport proteins. Recent studies have found that oxidized resveratrol (40 to 800 μM) dose-dependently reduces glucose transport, which seems to inhibit GLUT2 and SGLT1. Similar concentrations of Puag Haad also inhibited these transport proteins but with higher efficacy. Current therapies lack the ability to target glucose transporters in intestinal cells. Oxidized resveratrol and Puag Haad may provide new directions for inhibiting the function of transporters [52, 53]. However, the study on the mechanism of action of transporters is relatively lacking at present, and more in-depth studies should be conducted in the future, which may become a new way to control postprandial blood glucose.

#### 4.2.2 Intestinal Endocrine Cells Can Secrete Intestinal Proinsulin, such as GLP-1, to Regulate Blood Sugar Levels

In the past 20 years, a large number of articles have conducted in-depth studies on intestinal endocrine cells and incretin screened a group of incretin related to blood glucose regulation, especially GLP-1 and its related hormones, and successfully developed drugs such as liraglutide and semaglutide for clinical use. Since the introduction of Liraglutide and Semaglutide, they have had a good effect on T2DM, weight loss, inhibition of excessive appetite, *etc*. GLP-1 and its related fields are still a hot topic of research today. The intestinal endocrine cells (EECs) in intestinal cells can secrete a variety of hormones to help the body regulate energy balance [54-56]. Among them, the intestinal endocrine cells closely related to diabetes are K cells in the proximal small intestine (mainly the duodenum) and L cells in the jejunum, ileum, and colon. GLP-1 is secreted by intestinal endocrine L cells, mainly located in the distal ileum and colon. GIP is released from intestinal K cells located in the proximal region of the small intestine (duodenum and jejunum) [57]. After evaluating the intake of nutrients from food in the gastrointestinal tract, intestinal endocrine cells trigger the secretion of GLP-1 and GIP through sensing mechanisms such as electrotransporters, ion channel regulation, and nutrient-activated G protein-coupled receptors. A few minutes after the body ingests nutrients, GLP-1 and GIP begin to secrete to help quickly process the ingested nutrients and change feeding behavior [58-60]. Both of them act on pancreatic beta cells through structurally different but related receptors. The glucagon like peptide-1 receptor (GLP-1R) and glucose dependent insulinotropic peptide receptor (GIPR) are two B1 G protein coupled receptors, and their activation leads to increased secretion of cyclic adenosine monophosphate (cAMP) and glucose dependent insulin secretion β-cell proliferation induction and enhanced resistance to cell apoptosis [61, 62]. Specifically, GLP-1 can inhibit the secretion of glucagon by slowing gastric emptying and glucose dependent inhibition. In addition, GLP-1 can also promote satiety to reduce patient food intake, and regulate glucose through the above pathways [63]. Therefore, GIP may enhance GLP-1 function by increasing its population of pro-apoptotic neurons entering the medial basal nucleus of the hypothalamus. Therefore, regulating the release of endogenous storage of GLP-1 and GIP is considered a promising strategy with positive effects on patient weight, food intake, and blood glucose levels [64]. Among the existing therapeutic drugs, in addition to GLP-1 receptor agonists, dual GIP/GLP-1 receptor agonists are currently the latest research direction. It is known that the latest GIP and GLP-1 RA dual drug is tirzepatide, which has better dose dependence and efficacy in the SURPASS program compared to placebo, insulin, and two GLP-1 analogues [65-67]. In addition, some scholars have used glucose to stimulate GLUTag cells and human jejunal enteroids, focusing on cell signaling pathways. They found that βHB has an inhibitory effect on glucose-stimulated GLP-1 secretion, and this effect may open up new therapeutic pathways for promoting GLP-1 secretion in the future [68].

However, with the clinical application, the dose-related gastrointestinal effects of GLP-1 limit efficacy. The role of GIP in the central nervous system may provide further metabolic benefits by reducing energy expenditure, especially when used in combination with GLP-1, which can cause stronger anorexia and weight loss effects. These two drugs have complementary or usually cumulative effects [69, 70]. Scholars have found that [71] GIPR is expressed in non-neuronal cells in the medial basal part of the hypothalamus, including oligodendrocytes that regulate peripheral signal entry into ARC. In addition, some studies have pointed out that the increase in GIP concentration will increase the occurrence of cardiovascular events. However, recent literature shows that a high concentration of GIP is beneficial to cardiovascular disease. Therefore, the study on tirzepatide effecting cardiovascular disease may bring a definitive conclusion to this uncertain issue. In any case, the research of intestinal endocrine cells and GLP-1 and other related fields will continue to be a hot spot in the future.

### 4.3 Future Research Frontiers of Intestinal Cells and T2DM

#### 4.3.1 Lack of Short-Chain Fatty Acids Increases Intestinal Epithelial Stem Cell Count, Inhibits Differentiation, and Subsequently Leads to Metabolic Syndrome

As hot research in this field has successfully developed a number of effective drugs and successfully entered clinical use, there may be more accurate discussions in the future. Midgut epithelial stem cells appeared in 2023 (Fig. **5**), and short-chain fatty acids appeared in 2020, both of which have become part of the Top 32 hot keywords and belong to the #0 cluster. Starting from the origin of intestinal epithelial stem cells (ISCs), the role of short-chain fatty acids on ISCs may be a new focus of future research. ISCs are the primitive cells that differentiate all intestinal epithelial cells. The intestinal epithelium turnover occurs every 3-5 days, and this rapid metabolism is maintained by ISCs expressing Lgr5 [72]. Intestinal epithelial stem cells play an important role in the digestion and nutrient absorption of the body [73, 74]. With the significant improvement in people's quality of life, the diet structure continues to tilt towards a high-fat diet, and its exposed health risks are increasing year by year. Scholars have found that patients develop insulin resistance under sustained high-fat diet (HFD), which may be related to affecting the function and morphology of intestinal epithelium [75, 76]. With sufficient experimental research, it has been found that HFD fed mice have increased ISC counts in the small intestine and colon compared to mice on a normal diet and induced self-renewal. This result explains how HFD affects intestinal epithelium [77]. ISCs are the source of differentiation for intestinal endocrine cells. A diabetic diet is an effective means to prevent and control the occurrence and development of diabetes. Therefore, the secretion function of GIP/GLP-1 by intestinal endocrine cells is closely related to intestinal epithelial stem cells. Studies have shown that short-chain fatty acids (SCFA) can increase the differentiation markers of EEC and promote the differentiation of ISC into EEC, HFD can reduce the production of SCFA and inhibit the differentiation of EEC [78, 79]. Moreover, as T2DM is an inflammatory disease, interleukin receptor antagonists and salicylic acid can reduce blood sugar while improving the chronic low-grade inflammation of T2DM. SCFAs act on a variety of immune cells to regulate their differentiation, chemotaxis, recruitment, proliferation, and apoptosis, and at the same time, can reduce the production of interleukin-1 (IL-1), interleukin-6 (IL-6) and other pro-inflammatory factors. Therefore, a moderate increase in the intake of SCFA in the diabetic diet may become a new dietary choice for patients. In addition, GIP/GLP-1 drugs are exogenous, some patients have poor compliance, and there are certain dose-dependent adverse reactions, SCFA can also be used as a new target to promote endogenous GIP/GLP-1 secretion in future drug research and development, and reduce the occurrence of dose-dependent.

#### 4.3.2 Weight Loss Surgery Drives Intestinal Epithelial Stem Cells to Differentiate into Intestinal Endocrine Cells Through Bile Acid Signals, Thereby Regulating Blood Sugar Levels

In recent years, with the development of laparoscopy and the obesity problem brought by high-fat diet, bariatric surgery has gradually entered the field of vision of patients. In (Fig. **5**), both bile acids and sleeve gastrectomy first appeared in the 2022-2023 period, located in cluster #7, and have become the Top 32 hot keywords. Therefore, research on bile acids and sleeve gastrectomy may be a new hot spot in future research. So far, weight loss surgeries such as sleeve gastrectomy and gastric bypass surgery (RYGB) are still effective methods for sustainable weight loss and alleviating T2DM [80-84]. Scholars have found [85-87] that the number of intestinal endocrine cells secreting GLP-1 significantly increases after vertical sleeve gastrectomy (VSG), but in mouse models lacking the bile acid receptor, farnesol X receptor (FXR), the beneficial effects of VSG on EEC disappear [88-90]. With in-depth research, scholars conducted VSG on EGFP expressing mice in ISC [91] and RNA Seq on GFP positive cells sorted from intestinal epithelium. Subsequently, they also evaluated changes in the number of EECs after treatment with bile acids, FXR agonists and FXR antagonists. The RNA Seq of ISCs shows the expression of bile acid receptors in ISCs. Similarly, the levels of bile acids in mice treated with other weight loss surgeries, such as RYGB, were significantly increased and blood glucose levels were controlled [92]. This suggests that the pathway by which weight loss surgeries drive the differentiation of intestinal epithelial stem cells into intestinal endocrine cells may be due to bile acid signaling. Peptide tyrosine tyrosine (PYY) and GLP-1 are co-secreted by EEC L cells in the distal intestine, and PYY has been found to contribute to the release of GLP-1 and GIP [93-96]. By detecting L cells in the EEC of animal models, scholars have found that the increased secretion of PYY in the ileum after meals of RYGB may be one of the ways to improve T2DM after weight loss surgery [97, 98]. Although bariatric surgery is more effective than oral medications for obese T2DM patients, its treatment is not an option for most patients compared to oral medications. With the in-depth study of the mechanism of bariatric surgery, the role of bile acid signaling and PYY has been discovered, and future research may be inclined to it, and it may be used as a new drug method to replace bariatric surgery treatment in the future, so that more patients can easily accept it, providing a powerful intervention for T2DM.

### 4.4 Limitations

Firstly, due to the different economic levels of each country, this will inevitably affect the research process of each country. Secondly, this study only analyzed the English literature published in SCI-EXPANDED from 2004 to 2023 in WOS. The selection of literature libraries is not comprehensive enough, and there may be omissions in literature before 2004 and non-English literature. Future research can be combined with other literature libraries, such as CNKI, for joint research. Finally, despite strict manual screening and programmatic analysis, selection bias still exists due to the merging of certain keywords and database updates.

## CONCLUSION

In this study, CiteSpace, VOSviewer, the “Bibliometrix” R package, and Scimago Graphica softwares were used for the first time to conduct a bibliometric analysis of the visualization in the research field of intestinal cells and type 2 diabetes in WOS from 2004 to 2023. They evaluated the authors, countries, institutions, keywords, references, *etc*., in different years in a more efficient and intuitive way and analyzed the current research trends, progress and future development direction of this research field. The hot spots of research are “intestinal epithelial cells”, “GLP-1”, “SGLT-1”, “GLUT2” and “intestinal endocrine cell”, while “intestinal epithelial stem cells”, “short-chain fatty acid”, “bile acid” and “bariatric surgery” may be the focus of future research. We believe that this study can help clinicians to have a deeper understanding of type 2 diabetes and provide a research reference for scholars to study the relationship between intestinal cells and type 2 diabetes.

## Figures and Tables

**Fig. (1):**
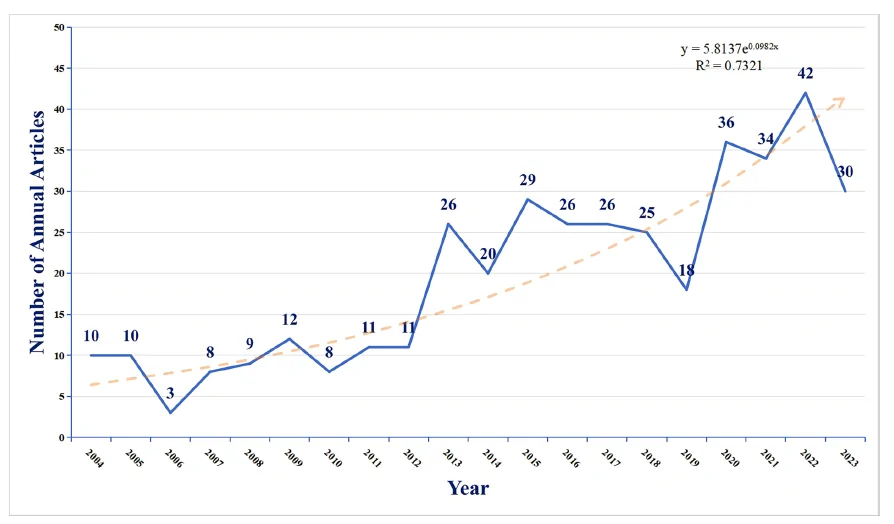
The number of annual articles has been steadily increasing from 1 January 2004 to 31 December 2023.

**Fig. (2):**
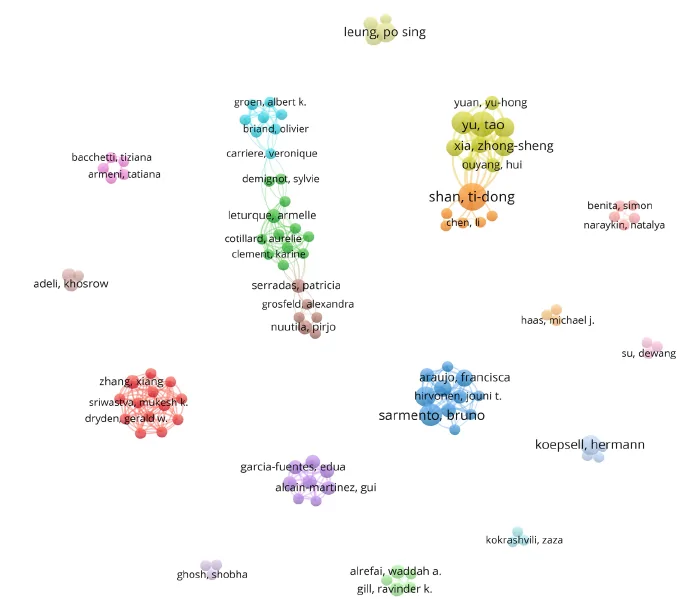
VOSviewer core author with Total Link Strength > 2 collaboration Overlay Visualization from 1 January 2004 to 31 December 2023.

**Fig. (3):**
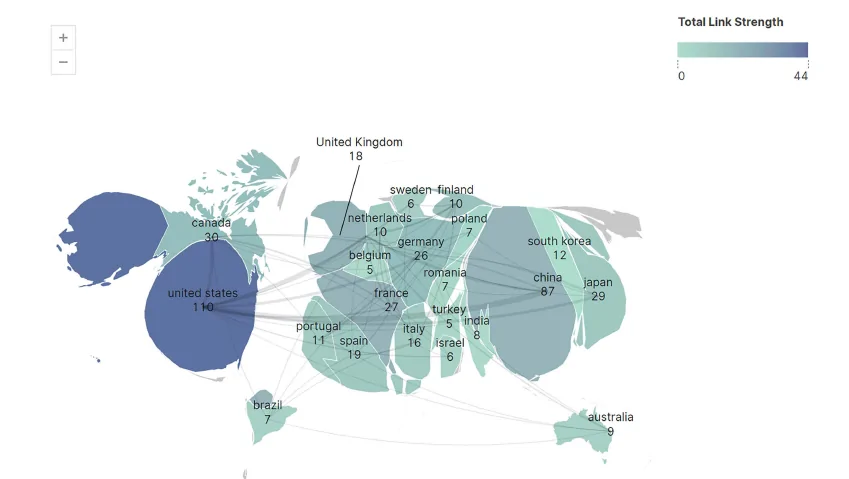
Visualize network of countries with national publications(N≥5) and the total link strength from 1 January 2004 to 31 December 2023.

**Fig. (4):**
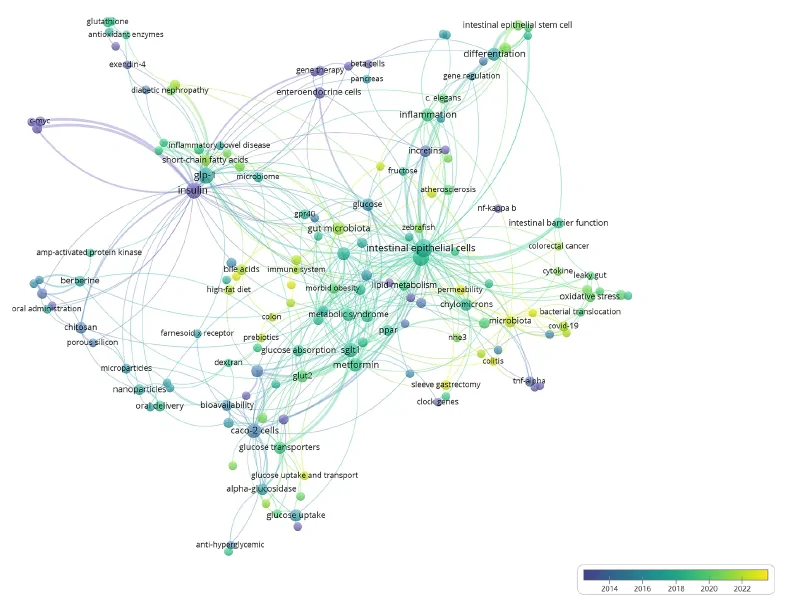
The author keywords (f>2) and the total link strength overlay the year for visualization research from 1 January 2004 to 31 December 2023.

**Fig. (5):**
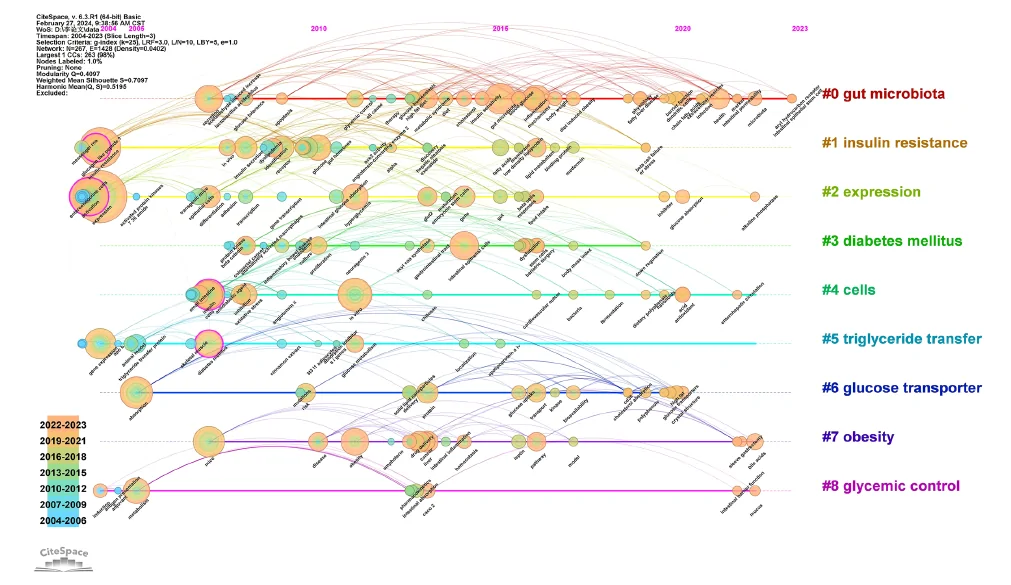
The timeline view map analysis of the Keywords and divided into 9 clusters by CiteSpace (Slice Length=3) from 1 January 2004 to 31 December 2023.

**Fig. (6):**
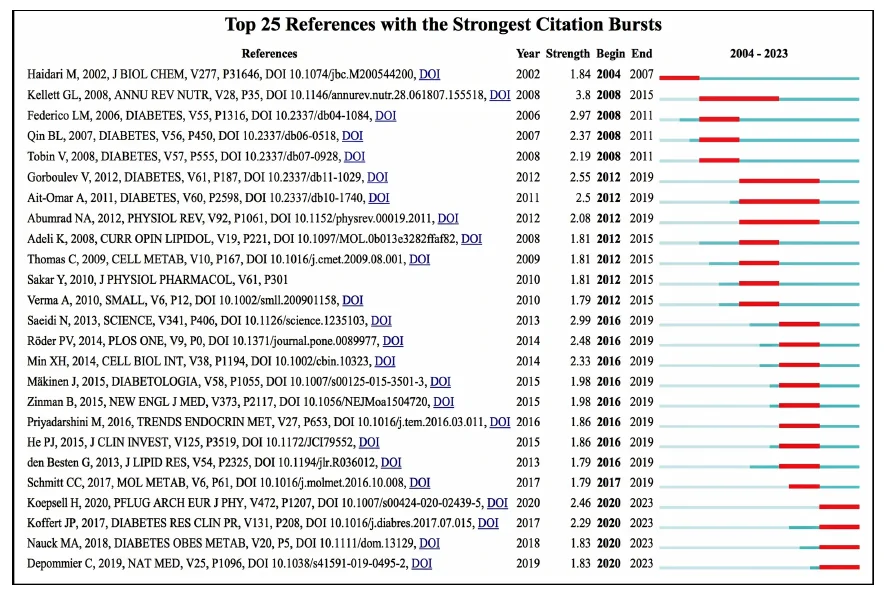
Top 25 references with the strongest citation bursts by CiteSpace.

**Fig. (7):**
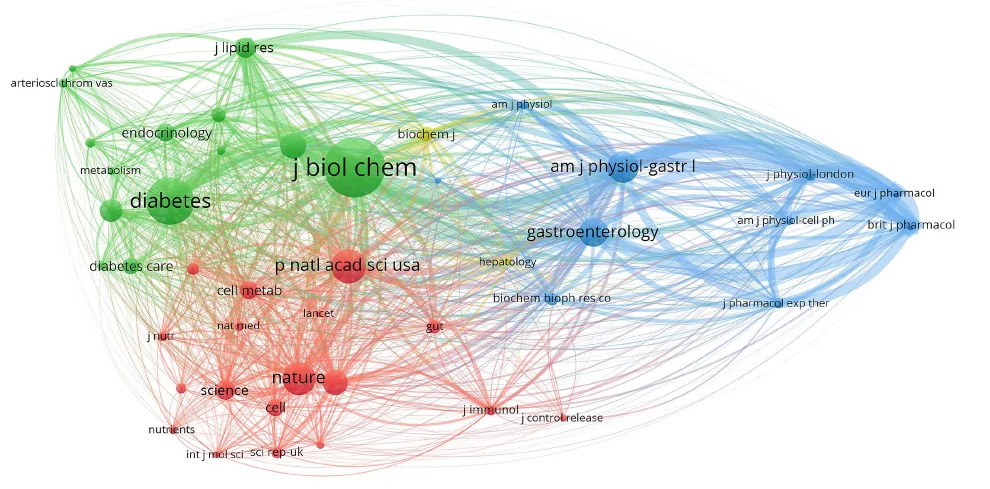
Co-citation network diagram with minimum co-citation equal to 95 and divided into 3 clusters.

**Fig. (8):**
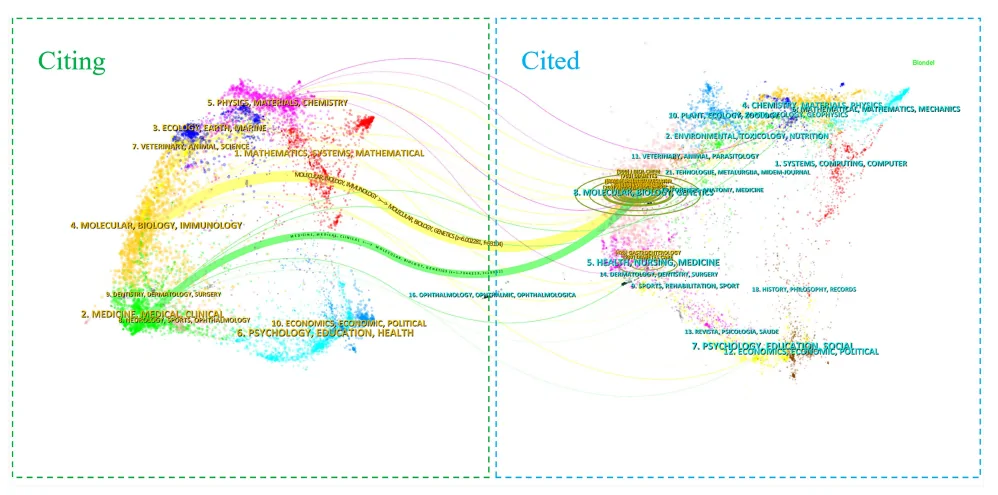
The dual-map overlay of journals on research. The left side is a cluster of cited journals, and the right side is a cluster of cited journals.

**Fig. (9):**
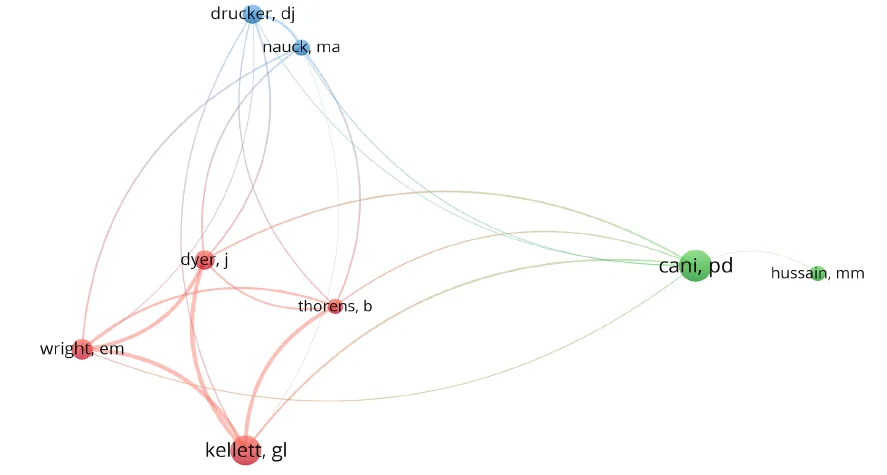
Co-citation authors analysis network map by setting the minimum equal of citation as 30 and dividing into 3 clusters.

**Table 1:** Most important authors in the Education big data research field.

Author	Countries	Publications	Citations (Citations Per Paper)	H-index	M-index
Ti-dong Shan	China	8	82(10.25)	5	0.71
Tao Yu	China	7	127(18.14)	7	0.53
Qi-kui Chen	China	6	117(19.5)	6	0.46
Bruno Sarmento	Portugal	6	522(87)	6	0.50
Zhong-sheng Xia	China	5	79(15.8)	4	0.44
Wa Zhong	China	5	110(22)	5	0.55
Neha Shrestha	Nepal	5	448(89.6)	5	0.55
Hermann Koepsell	Germany	5	257(51.4)	4	0.20
Po sing Leung	China	5	134(26.8)	5	0.31

**Table 2:** Top 10 countries and institutions in terms of number of publications.

Rank	Country(Continents)	Counts	Institutions	Counts
1	United States(North America)	110(27.9%)	University of Toronto(Canada)	11(2.7%)
2	China(Asia)	87(22%)	University of Porto(Portugal)	8(2%)
3	Canada(North America)	30(7.6%)	University of Turku(Finland)	8(2%)
4	Japan(Asia)	29(7.3%)	Sun yat sen university(China)	8(2%)
5	France(Europe)	27(6.8%)	University of Helsinki(Finland)	7(1.7%)
6	Germany(Europe)	26(6.6%)	University of Wurzburg(Germany)	7(1.7%)
7	Spain(Europe)	19(4.8%)	German center for diabetes research(Germany)	6(1.5%)
8	United Kingdom(Europe)	18(4.5%)	Technische Universität München(Germany)	6(1.5%)
9	Italy(Europe)	16(4%)	The Chinese University of Hong Kong(China)	6(1.5%)
10	South Korea(Asia)	12(3%)	Inserm(France)	6(1.5%)

**Table 3:** The top 32 keywords according to occurrence.

Rank	Keywords	Occurences	Total Link Strength	Rank	Keywords	Occurences	Total Link Strength
1	intestinal epithelial cells	33	29	17	glucose uptake	7	2
2	obesity	23	24	18	alpha-glucosidase	6	7
3	glp-1	20	15	19	chitosan	6	4
4	insulin	20	16	20	enteroendocrine cells	6	5
5	caco-2 cells	14	13	21	glucose absorption	6	5
6	sglt1	13	15	22	incretins	6	5
7	metformin	12	8	23	microbiota	6	5
8	differentiation	10	12	24	microrna	6	9
9	gut microbiota	10	6	25	bile acids	5	1
10	inflammation	10	8	26	intestinal epithelial stem cell	5	7
11	insulin resistance	10	9	27	lipid metabolism	5	5
12	chylomicrons	8	8	28	short-chain fatty acids	5	7
13	glucose transporters	8	8	29	bariatric surgery	4	8
14	glut2	8	13	30	intestinal barrier function	4	6
15	metabolic syndrome	8	4	31	berberine	4	5
16	glucose transport	7	8	32	ppar	4	5

**Table 4:** Top 5 references with citation bursts in order of strength.

Rank	Title	Author, Year	Strength	Journal	IF
1	Sugar absorption in the intestine: the role of GLUT2	George L Kellett,2008	3.8	Annual Review of Nutrition	8.9
2	Reprogramming of intestinal glucose metabolism and glycemic control in rats after gastric bypass	Nima Saeidi,2013	2.99	Science	56.9
3	Intestinal insulin resistance and aberrant production of apolipoprotein B48 lipoproteins in an animal model of insulin resistance and metabolic dyslipidemia: evidence for activation of protein tyrosine phosphatase-1B, extracellular signal-related kinase, and sterol regulatory element-binding protein-1c in the fructose-fed hamster intestine	Lisa M Federico,2006	2.97	Diabetes	7.77
4	Na(+)-D-glucose cotransporter SGLT1 is pivotal for intestinal glucose absorption and glucose-dependent incretin secretion	Valentin Gorboulev,2012	2.55	Diabetes	7.77
5	GLUT2 accumulation in enterocyte apical and intracellular membranes: a study in morbidly obese human subjects and ob/ob and high fat-fed mice	Amal Ait-Omar,2011	2.5	Diabetes	7.77

**Table 5:** The top 10 co-cited journals.

Rank	Co-cited Journal	IF	Q	Co-citations
1	The Journal of Biological Chemistry	4.8	Q2	900
2	Diabetes	7.7	Q1	703
3	Proceedings of the National Academy of Sciences of the United States of America	11.1	Q1	502
4	Nature	64.8	Q1	481
5	American Journal Of Physiology-gastrointestinal And Liver Physiology	4.5	Q1	461
6	Gastroenterology	29.4	Q1	428
7	Journal of Clinical Investigation	15.9	Q1	372
8	Plos One	3.7	Q2	366
9	Diabetologia	8.2	Q1	328
10	Journal of Lipid Research	6.5	Q1	295

## Data Availability

The data and supportive information are available within the article.

## LIST OF ABBREVIATIONS

WoSCC: Web of Science Core Collection
T2DM: Type 2 Diabetes Mellitus
RCT: Randomized Controlled Trial
GLUT: Glucose Transporters
ISCs: Intestinal Epithelial Stem Cells
EECs: Endocrine Cells

## References

[1] Dugani S.B., Mielke M.M., Vella A. (2021). Burden and management of type 2 diabetes in rural United States.. Diabetes Metab. Res. Rev..

[2] Wu X., Gao Y., Wang M., Peng H., Zhang D., Qin B., Pan L., Zhu G. (2023). Atherosclerosis indexes and incident T2DM in middle-aged and older adults: Evidence from a cohort study.. Diabetol. Metab. Syndr..

[3] Chew N.W.S., Ng C.H., Tan D.J.H., Kong G., Lin C., Chin Y.H., Lim W.H., Huang D.Q., Quek J., Fu C.E., Xiao J., Syn N., Foo R., Khoo C.M., Wang J.W., Dimitriadis G.K., Young D.Y., Siddiqui M.S., Lam C.S.P., Wang Y., Figtree G.A., Chan M.Y., Cummings D.E., Noureddin M., Wong V.W.S., Ma R.C.W., Mantzoros C.S., Sanyal A., Muthiah M.D. (2023). The global burden of metabolic disease: Data from 2000 to 2019.. Cell Metab..

[4] Song Y., Zhu C., Shi B., Song C., Cui K., Chang Z., Gao G., Jia L., Fu R., Dong Q., Feng L., Zhu C., Yin D., Manson J.E., Dou K. (2023). Social isolation, loneliness, and incident type 2 diabetes mellitus: Results from two large prospective cohorts in Europe and East Asia and Mendelian randomization.. EClinicalMedicine.

[5] Antza C., Kostopoulos G., Mostafa S., Nirantharakumar K., Tahrani A. (2022). The links between sleep duration, obesity and type 2 diabetes mellitus.. J. Endocrinol..

[6] Muzurović E., Mikhailidis D.P., Mantzoros C. (2021). Non-alcoholic fatty liver disease, insulin resistance, metabolic syndrome and their association with vascular risk.. Metabolism.

[7] Yun J.S., Ko S.H. (2021). Current trends in epidemiology of cardiovascular disease and cardiovascular risk management in type 2 diabetes.. Metabolism.

[8] Ma Q., Li Y., Li P., Wang M., Wang J., Tang Z., Wang T., Luo L., Wang C., Wang T., Zhao B. (2019). Research progress in the relationship between type 2 diabetes mellitus and intestinal flora.. Biomed Pharmacother..

[9] Arora P., Andersen D., Moll J.M., Danneskiold-Samsøe N.B., Xu L., Zhou B., Kladis G., Rausch P., Workman C.T., Kristiansen K., Brix S. (2021). Small intestinal tuft cell activity associates with energy metabolism in diet-induced obesity.. Front. Immunol..

[10] Roldan-Valadez E., Salazar-Ruiz S.Y., Ibarra-Contreras R., Rios C. (2019). Current concepts on bibliometrics: A brief review about impact factor, Eigenfactor score, CiteScore, SCImago Journal Rank, Source-Normalised Impact per Paper, H-index, and alternative metrics.. Ir. J. Med. Sci..

[11] Ninkov A., Frank J.R., Maggio L.A. (2021). Bibliometrics: Methods for studying academic publishing.. Perspect. Med. Educ..

[12] Chen C. (2004). Searching for intellectual turning points: Progressive knowledge domain visualization.. Proc. Natl. Acad. Sci. U.S.A..

[13] Synnestvedt M.B., Chen C., Holmes J.H. (2005). CiteSpace II: Visualization and knowledge discovery in bibliographic databases.. AMIA Annu Symp Proc..

[14] Arruda H., Silva E.R., Lessa M., Proença D., Bartholo R. (2022). VOSviewer and Bibliometrix.. J. Med. Libr. Assoc..

[15] Song Y., Wei K., Yang S., Shu F., Qiu J. (2023). Analysis on the research progress of library and information science since the new century.. Libr. Hi Tech.

[16] Mao Y., Zhao K., Chen N., Fu Q., Zhou Y., Kong C., Li P., Yang C. (2023). A 2-decade bibliometric analysis of epigenetics of cardiovascular disease: From past to present.. Clin. Epigenetics.

[17] Price D.J.S. (1965). Networks of scientific papers.. Science.

[18] Kawamura M., Thomas C.D.L., Tsurumoto A., Sasahara H., Kawaguchi Y. (2000). Lotka’s law and productivity index of authors in a scientific journal.. J. Oral Sci..

[19] Venable G.T., Shepherd B.A., Loftis C.M., McClatchy S.G., Roberts M.L., Fillinger M.E., Tansey J.B., Klimo P. (2016). Bradford’s law: Identification of the core journals for neurosurgery and its subspecialties.. J. Neurosurg..

[20] Hernández-Fernández A., Torre I.G. (2022). Compression principle and Zipf’s Law of brevity in infochemical communication.. Biol. Lett..

[21] Mayta-Tovalino F., Espinoza-Carhuancho F., Alvitez-Temoche D., Mauricio-Vilchez C., Munive-Degregori A., Barja-Ore J., Pacheco-Mendoza J. (2023). Dynamicity, emerging patterns, and spatiotemporal trends of scientific production on the use of activated carbon in oral health: A scientometric study.. BMC Oral Health.

[22] Jing Y., Wang C., Chen Z., Shen S., Shadiev R. (2024). A bibliometric analysis of studies on technology-supported learning environments: Hot topics and frontier evolution.. J. Comput. Assist. Learn..

[23] Jiang S.T., Liu Y.G., Zhang L., Sang X.T., Xu Y.Y., Lu X. (2022). Immune-related adverse events: A bibliometric analysis.. Front. Immunol..

[24] Sun H.L., Bai W., Li X.H., Huang H., Cui X.L., Cheung T., Su Z.H., Yuan Z., Ng C.H., Xiang Y.T. (2022). Schizophrenia and inflammation research: A bibliometric analysis.. Front. Immunol..

[25] Xu Y., Jiang Z., Kuang X., Chen X., Liu H. (2022). Research trends in immune checkpoint blockade for Melanoma: Visualization and bibliometric analysis.. J. Med. Internet Res..

[26] Zeng L., Ma G., Chen K., Zhou Q. (2023). Bibliometric analysis of rheumatic immune related adverse events associated with immune checkpoint inhibitors.. Front. Immunol..

[27] Zhang H., Shi Y., Ying J., Chen Y., Guo R., Zhao X., Jia L., Xiong J., Jiang F. (2023). A bibliometric and visualized research on global trends of immune checkpoint inhibitors related complications in melanoma, 2011–2021.. Front. Endocrinol..

[28] Fu X., Tan H., Huang L., Chen W., Ren X., Chen D. (2023). Gut microbiota and eye diseases: A bibliometric study and visualization analysis.. Front. Cell. Infect. Microbiol..

[29] Wei N., Xu Y., Li Y., Shi J., Zhang X., You Y., Sun Q., Zhai H., Hu Y. (2022). A bibliometric analysis of T cell and atherosclerosis.. Front. Immunol..

[30] Haidari M., Leung N., Mahbub F., Uffelman K.D., Kohen-Avramoglu R., Lewis G.F., Adeli K. (2002). Fasting and postprandial overproduction of intestinally derived lipoproteins in an animal model of insulin resistance. Evidence that chronic fructose feeding in the hamster is accompanied by enhanced intestinal *de novo* lipogenesis and ApoB48-containing lipoprotein overproduction.. J. Biol. Chem..

[31] Koepsell H. (2020). Glucose transporters in the small intestine in health and disease.. Pflugers Arch..

[32] Kellett G.L., Brot-Laroche E., Mace O.J., Leturque A. (2008). Sugar absorption in the intestine: The role of GLUT2.. Annu. Rev. Nutr..

[33] Federico L.M., Naples M., Taylor D., Adeli K. (2006). Intestinal insulin resistance and aberrant production of apolipoprotein B48 lipoproteins in an animal model of insulin resistance and metabolic dyslipidemia: Evidence for activation of protein tyrosine phosphatase-1B, extracellular signal-related kinase, and sterol regulatory element-binding protein-1c in the fructose-fed hamster intestine.. Diabetes.

[34] Ait-Omar A., Monteiro-Sepulveda M., Poitou C., Le Gall M., Cotillard A., Gilet J., Garbin K., Houllier A., Château D., Lacombe A., Veyrie N., Hugol D., Tordjman J., Magnan C., Serradas P., Clément K., Leturque A., Brot-Laroche E. (2011). GLUT2 accumulation in enterocyte apical and intracellular membranes: A study in morbidly obese human subjects and ob/ob and high fat-fed mice.. Diabetes.

[35] Gorboulev V., Schürmann A., Vallon V., Kipp H., Jaschke A., Klessen D., Friedrich A., Scherneck S., Rieg T., Cunard R., Veyhl-Wichmann M., Srinivasan A., Balen D., Breljak D., Rexhepaj R., Parker H.E., Gribble F.M., Reimann F., Lang F., Wiese S., Sabolic I., Sendtner M., Koepsell H. (2012). Na(+)-D-glucose cotransporter SGLT1 is pivotal for intestinal glucose absorption and glucose-dependent incretin secretion.. Diabetes.

[36] Saeidi N., Meoli L., Nestoridi E., Gupta N.K., Kvas S., Kucharczyk J., Bonab A.A., Fischman A.J., Yarmush M.L., Stylopoulos N. (2013). Reprogramming of intestinal glucose metabolism and glycemic control in rats after gastric bypass.. Science.

[37] Röder P.V., Geillinger K.E., Zietek T.S., Thorens B., Koepsell H., Daniel H. (2014). The role of SGLT1 and GLUT2 in intestinal glucose transport and sensing.. PLoS One.

[38] Chan L.K.Y., Leung P.S. (2015). Multifaceted interplay among mediators and regulators of intestinal glucose absorption: Potential impacts on diabetes research and treatment.. Am. J. Physiol. Endocrinol. Metab..

[39] Chen L., So W.Y., Li S.Y.T., Cheng Q., Boucher B.J., Leung P.S. (2015). Niacin-induced hyperglycemia is partially mediated *via* niacin receptor GPR109a in pancreatic islets.. Mol. Cell. Endocrinol..

[40] Baraille F., Ayari S., Carrière V., Osinski C., Garbin K., Blondeau B., Guillemain G., Serradas P., Rousset M., Lacasa M., Cardot P., Ribeiro A. (2015). Glucose tolerance is improved in mice invalidated for the nuclear receptor HNF-4γ: A critical role for enteroendocrine cell lineage.. Diabetes.

[41] Azevedo C., Pinto S., Benjakul S., Nilsen J., Santos H.A., Traverso G., Andersen J.T., Sarmento B. (2021). Prevention of diabetes-associated fibrosis: Strategies in FcRn-targeted nanosystems for oral drug delivery.. Adv. Drug Deliv. Rev..

[42] Górski A., Zimecki M., Krotkiewski H. (2021). Journal impact factor and self-citations.. Arch. Immunol. Ther. Exp..

[43] Moussa S. (2023). A bibliometric investigation of the journals that were repeatedly suppressed from Clarivate’s Journal Citation Reports.. Account. Res..

[44] Cani P.D., Dewever C., Delzenne N.M. (2004). Inulin-type fructans modulate gastrointestinal peptides involved in appetite regulation (glucagon-like peptide-1 and ghrelin) in rats.. Br. J. Nutr..

[45] Cani P.D., Daubioul C.A., Reusens B., Remacle C., Catillon G., Delzenne N.M. (2005). Involvement of endogenous glucagon-like peptide-1(7–36) amide on glycaemia-lowering effect of oligofructose in streptozotocin-treated rats.. J. Endocrinol..

[46] Cani P.D., Hoste S., Guiot Y., Delzenne N.M. (2007). Dietary non-digestible carbohydrates promote L-cell differentiation in the proximal colon of rats.. Br. J. Nutr..

[47] Cani P.D., Holst J.J., Drucker D.J., Delzenne N.M., Thorens B., Burcelin R., Knauf C. (2007). GLUT2 and the incretin receptors are involved in glucose-induced incretin secretion.. Mol. Cell. Endocrinol..

[48] Sala-Rabanal M., Hirayama B.A., Ghezzi C., Liu J., Huang S.C., Kepe V., Koepsell H., Yu A., Powell D.R., Thorens B., Wright E.M., Barrio J.R. (2016). Revisiting the physiological roles of SGLTs and GLUTs using positron emission tomography in mice.. J. Physiol..

[49] Yu L.C.H., Huang C., Kuo W., Sayer H., Turner J.R., Buret A.G. (2008). SGLT-1-mediated glucose uptake protects human intestinal epithelial cells against Giardia duodenalis-induced apoptosis.. Int. J. Parasitol..

[50] Han L., Qu Q., Aydin D., Panova O., Robertson M.J., Xu Y., Dror R.O., Skiniotis G., Feng L. (2022). Structure and mechanism of the SGLT family of glucose transporters.. Nature.

[51] Maccari R., Ottanà R. (2022). Sodium-glucose cotransporter inhibitors as antidiabetic drugs: Current development and future perspectives.. J. Med. Chem..

[52] Wongon M., Limpeanchob N. (2020). Inhibitory effect of *Artocarpus lakoocha* Roxb and oxyresveratrol on α-glucosidase and sugar digestion in Caco_2_ cells.. Heliyon.

[53] Wongon M., Limpeanchob N. (2021). Artocarpus lacucha extract and oxyresveratrol inhibit glucose transporters in human intestinal Caco-2 cells.. Planta Med..

[54] Deacon C.F. (2005). What do we know about the secretion and degradation of incretin hormones?. Regul. Pept..

[55] Gribble F.M., Reimann F. (2016). Enteroendocrine cells: Chemosensors in the intestinal Epithelium.. Annu. Rev. Physiol..

[56] Beumer J., Puschhof J., Bauzá-Martinez J., Martínez-Silgado A., Elmentaite R., James K.R., Ross A., Hendriks D., Artegiani B., Busslinger G.A., Ponsioen B., Andersson-Rolf A., Saftien A., Boot C., Kretzschmar K., Geurts M.H., Bar-Ephraim Y.E., Pleguezuelos-Manzano C., Post Y., Begthel H., van der Linden F., Lopez-Iglesias C., van de Wetering W.J., van der Linden R., Peters P.J., Heck A.J.R., Goedhart J., Snippert H., Zilbauer M., Teichmann S.A., Wu W., Clevers H. (2020). High-resolution mRNA and secretome atlas of human enteroendocrine cells.. Cell.

[57] Mortensen K., Christensen L.L., Holst J.J., Orskov C. (2003). GLP-1 and GIP are colocalized in a subset of endocrine cells in the small intestine.. Regul. Pept..

[58] Holst J.J., Rosenkilde M.M. (2020). GIP as a therapeutic target in diabetes and obesity: Insight from incretin co-agonists.. J. Clin. Endocrinol. Metab..

[59] Lu V.B., Gribble F.M., Reimann F. (2021). Nutrient-induced cellular mechanisms of gut hormone secretion.. Nutrients.

[60] Nauck M.A., Müller T.D. (2023). Incretin hormones and type 2 diabetes.. Diabetologia.

[61] Campbell J.E., Newgard C.B. (2021). Mechanisms controlling pancreatic islet cell function in insulin secretion.. Nat. Rev. Mol. Cell Biol..

[62] Mayendraraj A., Rosenkilde M.M., Gasbjerg L.S. (2022). GLP-1 and GIP receptor signaling in beta cells – A review of receptor interactions and co-stimulation.. Peptides.

[63] Baggio L.L., Drucker D.J. (2007). Biology of Incretins: GLP-1 and GIP.. Gastroenterology.

[64] Gribble F.M., Reimann F. (2019). Function and mechanisms of enteroendocrine cells and gut hormones in metabolism.. Nat. Rev. Endocrinol..

[65] Frías J.P. (2020). Tirzepatide: A glucose-dependent insulinotropic polypeptide (GIP) and glucagon-like peptide-1 (GLP-1) dual agonist in development for the treatment of type 2 diabetes.. Expert Rev. Endocrinol. Metab..

[66] De Block C.E.M., Dirinck E., Verhaegen A., Van Gaal L.F. (2022). Efficacy and safety of high-dose glucagon-like peptide-1, glucagon-like peptide-1/glucose-dependent insulinotropic peptide, and glucagon-like peptide-1/glucagon receptor agonists in type 2 diabetes.. Diabetes Obes. Metab..

[67] Nauck M.A., D’Alessio D.A. (2022). Tirzepatide, a dual GIP/GLP-1 receptor co-agonist for the treatment of type 2 diabetes with unmatched effectiveness regrading glycaemic control and body weight reduction.. Cardiovasc. Diabetol..

[68] Elebring E., Casselbrant A., Persson S.M.T., Fändriks L., Wallenius V. (2023). βHB inhibits glucose-induced GLP-1 secretion in GLUTag and human jejunal enteroids.. J. Mol. Endocrinol..

[69] Bailey C.J. (2020). GIP analogues and the treatment of obesity-diabetes.. Peptides.

[70] Thondam S.K., Cuthbertson D.J., Wilding J.P.H. (2020). The influence of Glucose-dependent Insulinotropic Polypeptide (GIP) on human adipose tissue and fat metabolism: Implications for obesity, type 2 diabetes and non-alcoholic fatty liver disease (NAFLD).. Peptides.

[71] Samms R.J., Coghlan M.P., Sloop K.W. (2020). How may GIP enhance the therapeutic efficacy of GLP-1?. Trends Endocrinol. Metab..

[72] Sei Y., Feng J., Samsel L., White A., Zhao X., Yun S., Citrin D., McCoy J.P., Sundaresan S., Hayes M.M., Merchant J.L., Leiter A., Wank S.A. (2018). Mature enteroendocrine cells contribute to basal and pathological stem cell dynamics in the small intestine.. Am. J. Physiol. Gastrointest. Liver Physiol..

[73] Di W., Lv Y., Xia F., Sheng Y., Liu J., Ding G. (2020). Improvement of intestinal stem cells and barrier function *via* energy restriction in middle-aged C57BL/6 mice.. Nutr. Res..

[74] Strilbytska O.M., Storey K.B., Lushchak O.V. (2020). TOR signaling inhibition in intestinal stem and progenitor cells affects physiology and metabolism in Drosophila.. Comp. Biochem. Physiol. B Biochem. Mol. Biol..

[75] Rohr M.W., Narasimhulu C.A., Rudeski-Rohr T.A., Parthasarathy S. (2020). Negative effects of a high-fat diet on intestinal permeability: A review.. Adv. Nutr..

[76] Kang Y., Kang X., Yang H., Liu H., Yang X., Liu Q., Tian H., Xue Y., Ren P., Kuang X., Cai Y., Tong M., Li L., Fan W. (2022). *Lactobacillus acidophilus ameliorates* obesity in mice through modulation of gut microbiota dysbiosis and intestinal permeability.. Pharmacol. Res..

[77] Xie Y., Ding F., Di W., Lv Y., Xia F., Sheng Y., Yu J., Ding G. (2020). Impact of a high-fat diet on intestinal stem cells and epithelial barrier function in middle-aged female mice.. Mol. Med. Rep..

[78] Pearce S.C., Weber G.J., van Sambeek D.M., Soares J.W., Racicot K., Breault D.T. (2020). Intestinal enteroids recapitulate the effects of short-chain fatty acids on the intestinal epithelium.. PLoS One.

[79] Meyer R.K., Lane A.I., Weninger S.N., Martinez T.M., Kangath A., Laubitz D., Duca F.A. (2022). Oligofructose restores postprandial short-chain fatty acid levels during high-fat feeding.. Obesity.

[80] Jackson H.T., Anekwe C., Chang J., Haskins I.N., Stanford F.C. (2019). The role of bariatric surgery on diabetes and diabetic care compliance.. Curr. Diab. Rep..

[81] Courcoulas A.P., Gallagher J.W., Neiberg R.H., Eagleton E.B., DeLany J.P., Lang W., Punchai S., Gourash W., Jakicic J.M. (2020). Bariatric surgery *vs* lifestyle intervention for diabetes treatment: 5-year outcomes from a randomized trial.. J. Clin. Endocrinol. Metab..

[82] Chumakova-Orin M., Vanetta C., Moris D.P., Guerron A.D. (2021). Diabetes remission after bariatric surgery.. World J. Diabetes.

[83] Ji Y., Lee H., Kaura S., Yip J., Sun H., Guan L., Han W., Ding Y. (2021). Effect of bariatric surgery on metabolic diseases and underlying mechanisms.. Biomolecules.

[84] Sandoval D.A., Patti M.E. (2023). Glucose metabolism after bariatric surgery: Implications for T2DM remission and hypoglycaemia.. Nat. Rev. Endocrinol..

[85] Li F., Peng Y., Zhang M., Yang P., Qu S. (2016). Sleeve gastrectomy activates the GLP-1 pathway in pancreatic β cells and promotes GLP-1-expressing cells differentiation in the intestinal tract.. Mol. Cell. Endocrinol..

[86] Hong J., Bo T., Xi L., Xu X., He N., Zhan Y., Li W., Liang P., Chen Y., Shi J., Li D., Yan F., Gu W., Wang W., Liu R., Wang J., Wang Z., Ning G. (2021). Reversal of functional brain activity related to gut microbiome and hormones after vsg surgery in patients with obesity.. J. Clin. Endocrinol. Metab..

[87] Ribeiro-Parenti L., Jarry A.C., Cavin J.B., Willemetz A., Le Beyec J., Sannier A., Benadda S., Pelletier A.L., Hourseau M., Léger T., Morlet B., Couvelard A., Anini Y., Msika S., Marmuse J.P., Ledoux S., Le Gall M., Bado A. (2021). Bariatric surgery induces a new gastric mucosa phenotype with increased functional glucagon-like peptide-1 expressing cells.. Nat. Commun..

[88] Scott F., Elahi S., Adebibe M., Parampalli U., Mannur K., Góralczyk A., Sanger G.J. (2019). Farnesoid X receptor - A molecular predictor of weight loss after vertical sleeve gastrectomy?. Obes. Sci. Pract..

[89] Ding L., Zhang E., Yang Q., Jin L., Sousa K.M., Dong B., Wang Y., Tu J., Ma X., Tian J., Zhang H., Fang Z., Guan A., Zhang Y., Wang Z., Moore D.D., Yang L., Huang W. (2021). Vertical sleeve gastrectomy confers metabolic improvements by reducing intestinal bile acids and lipid absorption in mice.. Proc. Natl. Acad. Sci. USA.

[90] Tu J., Wang Y., Jin L., Huang W. (2022). Bile acids, gut microbiota and metabolic surgery.. Front. Endocrinol..

[91] Kim K.S., Peck B.C.E., Hung Y.H., Koch-Laskowski K., Wood L., Dedhia P.H., Spence J.R., Seeley R.J., Sethupathy P., Sandoval D.A. (2022). Vertical sleeve gastrectomy induces enteroendocrine cell differentiation of intestinal stem cells through bile acid signaling.. JCI Insight.

[92] Li K., Zou J., Li S., Guo J., Shi W., Wang B., Han X., Zhang H., Zhang P., Miao Z., Li Y. (2020). Farnesoid X receptor contributes to body weight-independent improvements in glycemic control after Roux-en-Y gastric bypass surgery in diet-induced obese mice.. Mol. Metab..

[93] Camacho-Ramírez A., Prada-Oliveira J.A., Ribelles-García A., Almorza-Gomar D., Pérez-Arana G.M. (2020). The leading role of peptide tyrosine tyrosine in glycemic control after Roux-en-Y gastric bypass in rats.. Obes. Surg..

[94] Boland B.B., Laker R.C., O’Brien S., Sitaula S., Sermadiras I., Nielsen J.C., Barkholt P., Roostalu U., Hecksher-Sørensen J., Sejthen S.R., Thorbek D.D., Suckow A., Burmeister N., Oldham S., Will S., Howard V.G., Gill B.M., Newton P., Naylor J., Hornigold D.C., Austin J., Lantier L., McGuinness O.P., Trevaskis J.L., Grimsby J.S., Rhodes C.J. (2022). Peptide-YY_3-36_/glucagon-like peptide-1 combination treatment of obese diabetic mice improves insulin sensitivity associated with recovered pancreatic β-cell function and synergistic activation of discrete hypothalamic and brainstem neuronal circuitries.. Mol. Metab..

[95] Jones L.A., Sun E.W., Lumsden A.L., Thorpe D.W., Peterson R.A., De Fontgalland D., Sposato L., Rabbitt P., Hollington P., Wattchow D.A., Keating D.J. (2023). Alterations in GLP-1 and PYY release with aging and body mass in the human gut.. Mol. Cell. Endocrinol..

[96] Xu J., Wang S., Wu H., Chen D., Han J., Lin Q. (2023). Engineering a potent and long-acting GLP-1/Y_2_ receptor dual agonist as a multi-agonist therapy for diabetes and obesity.. Peptides.

[97] Dischinger U., Corteville C., Otto C., Fassnacht M., Seyfried F., Hankir M. K. (2019). GLP-1 and PYY3-36 reduce high-fat food preference additively after Roux-en-Y gastric bypass in diet-induced obese rats.. Surg Obes Relat Dis..

[98] Pérez-Arana G.M., Díaz-Gómez A., Camacho-Ramírez A., Ribelles-García A., Almorza-Gomar D., Gracia-Romero M., Prada-Oliveira J.A. (2022). Peptide Tyrosine-Tyrosine triggers GLP-2-mediated intestinal hypertrophy after Roux-en-Y gastric bypass.. Obes. Surg..

