# Supplementary material for: Global Trends in Research of Intestinal Cells and Type 2 Diabetes Mellitus: A Bibliometric and Visualized Analysis from 2004 to 2023
Source: Endocr Metab Immune Disord Drug Targets. 2025 Apr 4;26:E18715303353611. doi: 10.2174/0118715303353611250312043509 (PMC13631935; doi:10.2174/0118715303353611250312043509)
Supplement: Supplementary file 1 [file EMIDDT-26-E18715303353611_SD1.pdf]

Supplementary Material

Global Trends in Research of Intestinal Cells and Type 2 Diabetes Mellitus: A Bibliometric and Visualized Analysis from 2004 to 2023

Zhaoyang Li[1,#], Shuang Zhang[2,#], Rufan Cao[1], Bo Wu[3], Kangning Han[1], Wenhui Yang[1], Shiyao Chen[4], Xinyu Zhang[1], Xiaochun Han[5,*] and Liangqing Guo[6,*]

[1]The First Clinical Medical College, Shandong University of Traditional Chinese Medicine, Jinan, 250355, China; [2]College of Foreign Language, Shandong University of Traditional Chinese Medicine, Jinan, 250355, China; [3]Department of Cardiovascular, Affiliated Hospital of Shandong University of Traditional Chinese Medicine, Jinan, 250011, China; [4]College of Traditional Chinese Medicine, Shandong University of Traditional Chinese Medicine, Jinan, 250355, China; [5]College of Health, Shandong University of Traditional Chinese Medicine, Jinan, 250355, China; [6]Department of Endocrinology, Affiliated Hospital of Shandong University of Traditional Chinese Medicine, Jinan, 250011, China

Table S1. Author productivity (Lotka's law).

Documents written	N. of Authors	Proportion of Authors(%)
1	2334	93.88
2	116	4.66
3	22	0.88
4	5	0.20
5	5	0.20
6	2	0.08
7	1	0.04
8	1	0.04

Supplementary Table 2.

Zone	Nº journals (%)		Number articles (%)		Bradford multipliers	
CORE	36	(14%)	131	(33%)		
Zone 1	94	(36%)	131	(33%)	1.00	
Zone 2	132	(50%)	132	(33%)	1.00	
Total	262	100%	394	100%	Mean	1.0

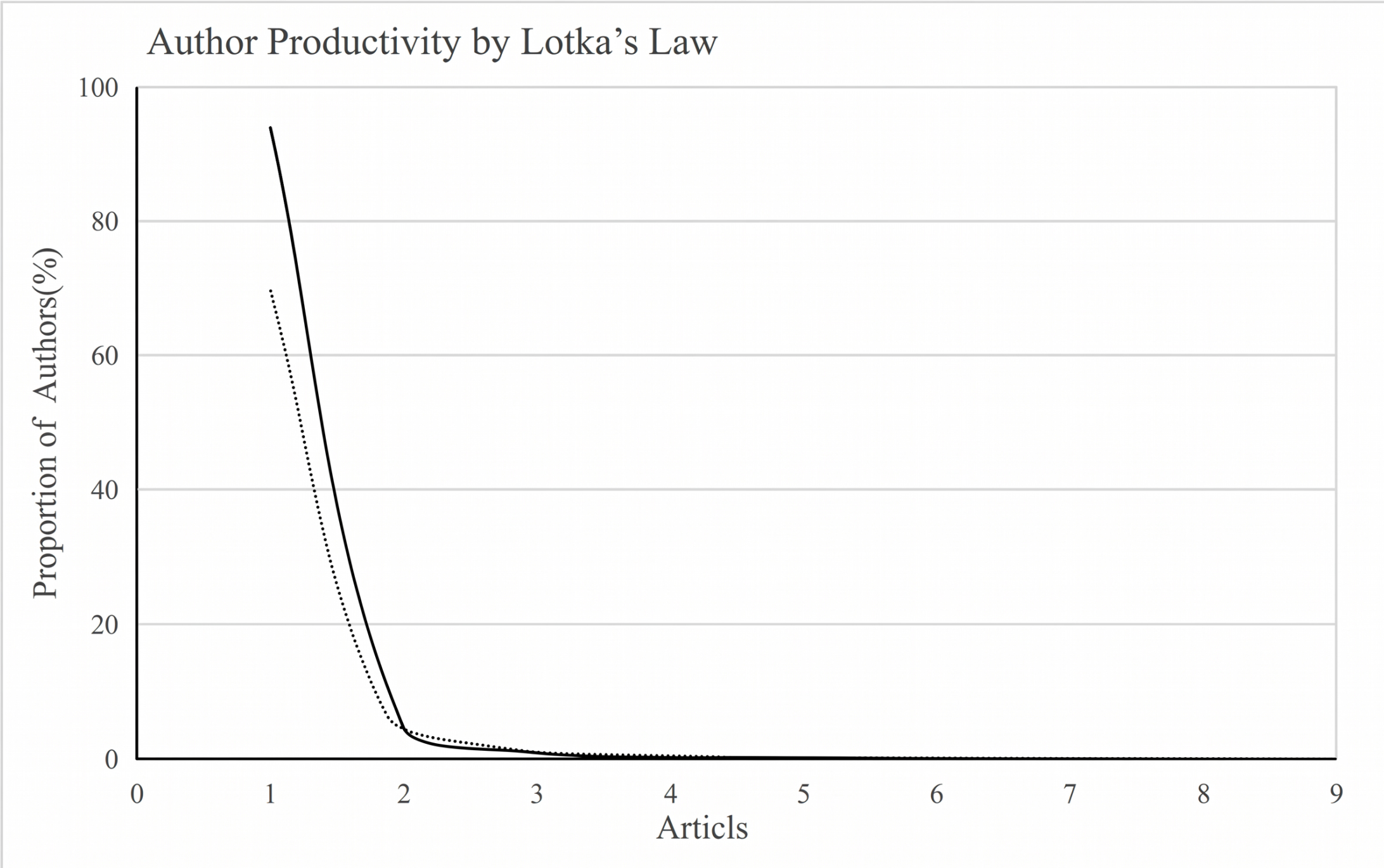
Author Productivity by Lotka's Law
Proportion of Authors(%)
100
80
60
40
20
0
0
1
2
3
4
5
6
7
8
9
Articls

Fig. (S1)

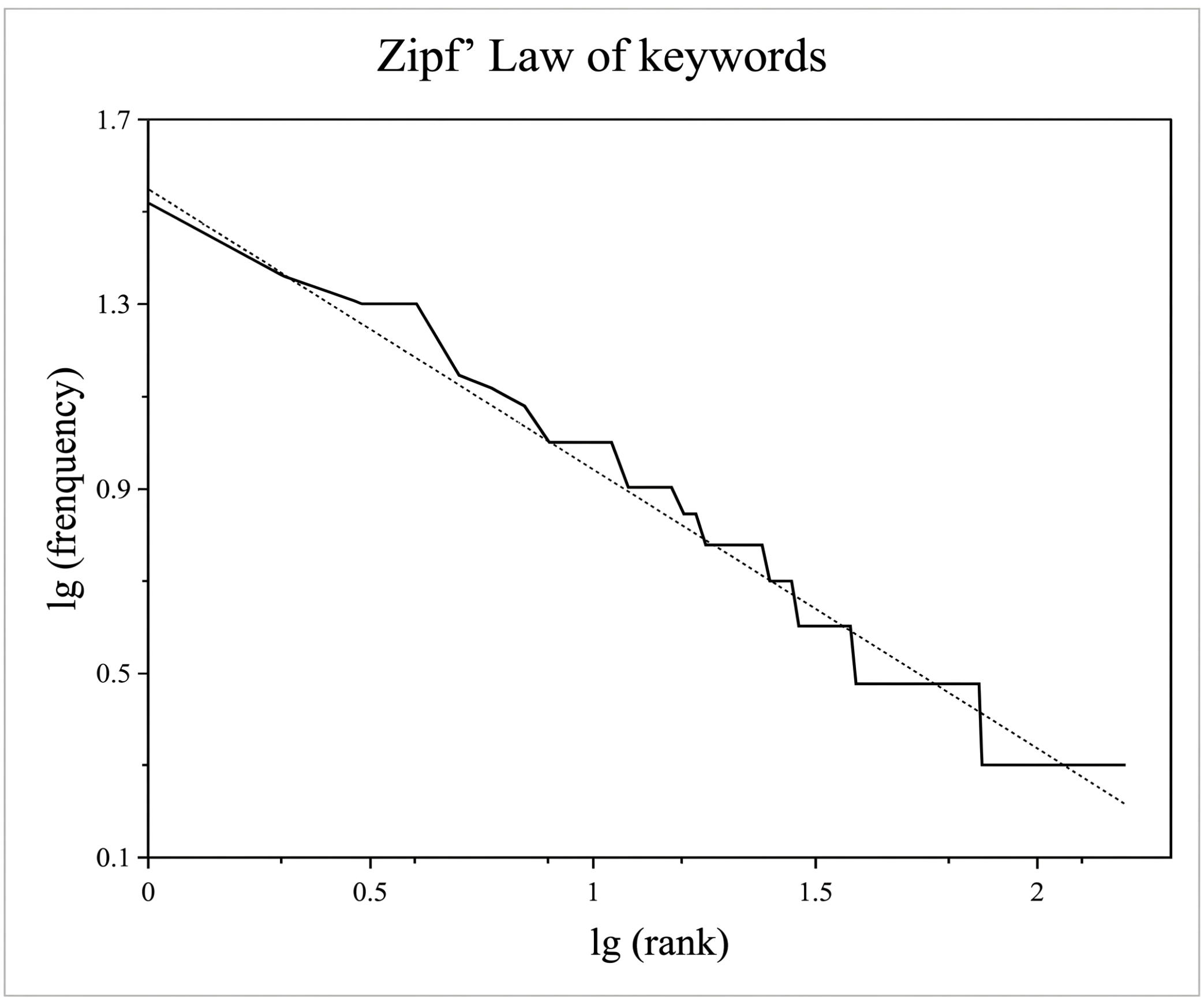
Zipf' Law of keywords
lg (frenquency)
1.7
1.3
0.9
0.5
0.1
0
0.5
1
1.5
2
lg (rank)

Fig. (S2)

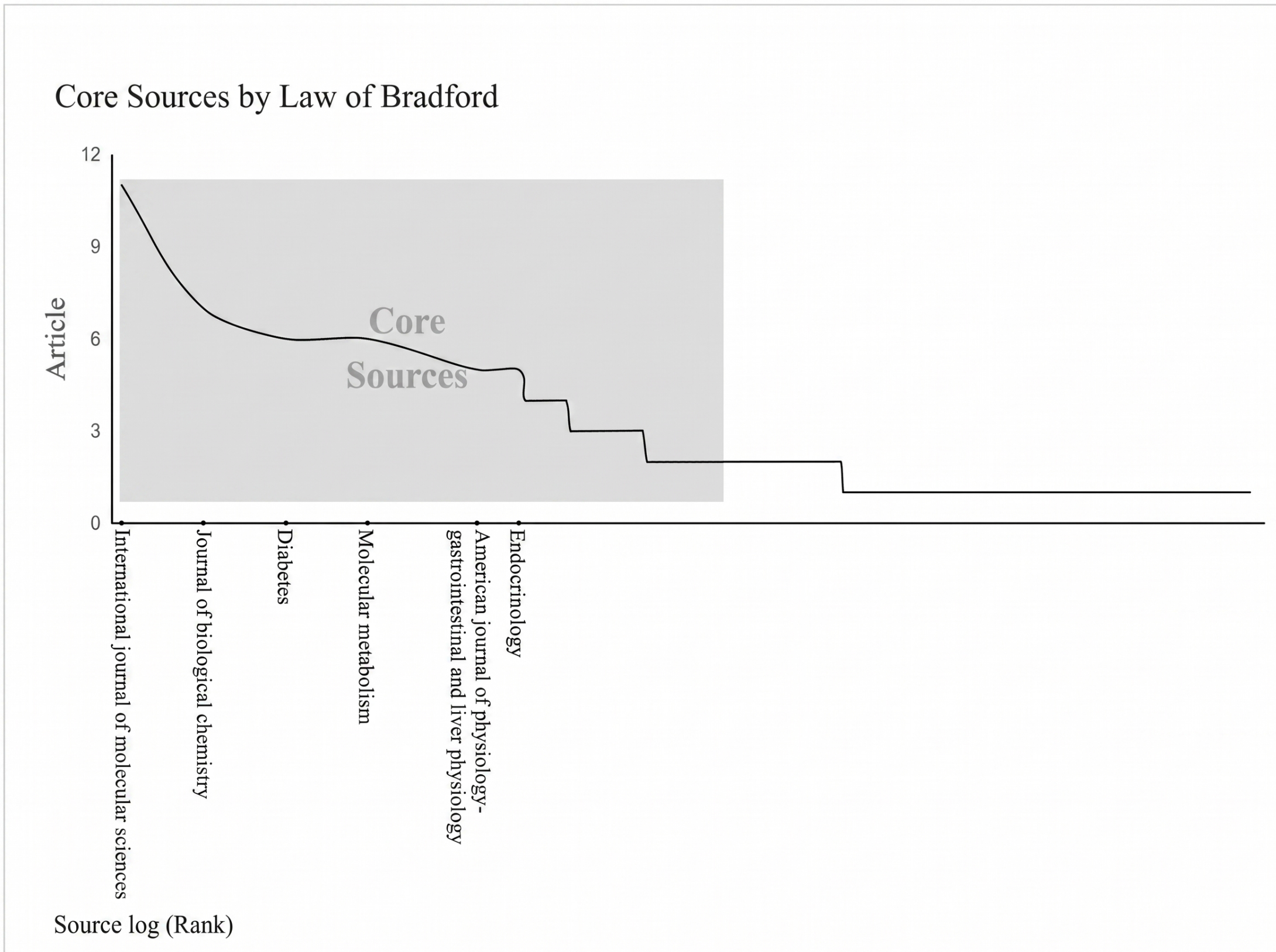
Core Sources by Law of Bradford
12
9
6
3
0
Article
Core
Sources
International journal of molecular sciences
Journal of biological chemistry
Diabetes
Molecular metabolism
American journal of physiology-gastrointestinal and liver physiology
Endocrinology
Source log (Rank)

Fig. (S3)
